# Mental health problems in a large representative sample of children and adolescents: Prevalence, associations with problematic substance use and psychometric properties of the Strengths and Difficulties Questionnaire

**DOI:** 10.3934/publichealth.2026042

**Published:** 2026-07-27

**Authors:** Lutz Wartberg, Rainer Thomasius, Nicolas Arnaud

**Affiliations:** 1 Department Psychology, Faculty of Human Sciences, MSH Medical School Hamburg, Hamburg, Germany; 2 German Center for Addiction Research in Childhood and Adolescence (DZSKJ), University Medical Center Hamburg-Eppendorf, Hamburg, Germany

**Keywords:** mental health, mental disorder, psychopathology, prevalence, SDQ, reliability, factor structure, alcohol, tobacco, cannabis

## Abstract

Mental health problems are already widespread in childhood and adolescence. The Strengths and Difficulties Questionnaire (SDQ) is an internationally widely used instrument for assessing mental health problems and prosocial behavior in children and adolescents. Particularly, the Total Difficulties score (TDS) from the SDQ (calculated from the sum of all problem scales) is considered a good estimator for mental health problems. Heretofore, there are relatively few estimates for mental health problems based on representative samples that rely on self-assessments by children and adolescents. Although SDQ is frequently used internationally, the findings regarding its psychometric properties are inconsistent. So far, there are no results available from representative samples on the relationships between the TDS and various forms of problematic substance use. A representative sample of 4001 12–17-year-olds from Germany was examined using the SDQ with regard to mental health problems and established standardized instruments for problematic use of alcohol, cigarettes, and cannabis. We calculated relative frequencies, reliability coefficients (Cronbach's alpha and McDonald's omega), correlations, binary logistic regressions, and linear regression analyses. Furthermore, we conducted confirmatory factor analyses. Based on the TDS, 9.6% (95% confidence interval: 8.7%–10.5%) of the representative sample reported severe mental health problems. We observed reliability values for the five SDQ scales between 0.44 (peer problems) and 0.70 (emotional problems), as well as 0.74 and 0.75 for the TDS. In the different confirmatory factor analyses, no model showed a completely satisfactory fit to the data. In bivariate and multivariable analyses, consistent statistically significant associations were found between mental health problems (TDS) and problematic cigarette use and problematic cannabis use, whereas findings for problematic alcohol use were mixed. According to our results, a substantial proportion of German children and adolescents are affected by severe mental health problems, and problematic substance use is a possible source of this.

## 1. Introduction

Mental health problems are already widespread in childhood and adolescence [1]. According to Goodman and Goodman [2], the Strengths and Difficulties Questionnaire (SDQ) “...is a measure of mental health problems in children aged 4 to 17 years...” (p. 401) that is internationally widely used [3]. The SDQ measures difficulties using four scales (emotional symptoms, conduct problems, hyperactivity, and peer relationship problems) and strengths using a further scale (prosocial behavior) [4]. In particular, the Total Difficulties score (TDS) from the SDQ (calculated from the sum of all four problem scales) is considered a good estimator for mental health problems [2]. The SDQ (and, thus, also the TDS) can be estimated both through self-assessment by older children and adolescents and through external assessment by parents or teachers (especially for younger children, but also for youth) [5]. Although many studies have been conducted with the SDQ, relatively few prevalence estimates exist for mental health problems based on representative samples that rely on self-assessments by children and adolescents.

Van Roy et al. [6] found a prevalence of 8.7% for abnormal mental health problems based on the TDS (range: 0–40, cutoff value ≥19) of the SDQ in the self-assessment of a representative sample of 10–19-year-olds from Norway. Woerner et al. [7] reported prevalence estimates for severe mental health problems for a representative sample of 11–16-year-olds from Thailand. Based on the TDS, the prevalence was 8.9% (cutoff value ≥19) according to the youths' self-rating, 8.6% according to the parents' assessment, and 9.8% according to the teachers' assessment [7]. From a representative sample of 11–17-year-olds from Germany, Becker et al. [8] observed a prevalence of severe mental health problems of 8.8% (cutoff value ≥17) based on the TDS self-assessment of children and adolescents. In a representative sample of Australian minors aged 11–17 years, Gray et al. [9] showed a prevalence of 10.2% (cutoff value ≥17) for severe mental health problems in self-rating with TDS and 9.4% with the parents' assessment. In Bezborodovs et al. [10], based on self-assessment (TDS) in a representative sample of 11–15-year-olds from Latvia, prevalence estimates of 9.4% (Latvian-speaking subsample) and 8.7% (Russian-speaking subsample) were found for severe mental health problems (cutoff values ≥22).

Although the SDQ is widely used internationally, findings regarding its psychometric properties (e.g., reliability) are not consistent, particularly with regard to the factor structure of the SDQ, with heterogeneous results [11]. The following results on the reliability of the SDQ in self-assessment were reported in representative samples. Goodman [12] found reliability coefficients (Cronbach's alpha) for the five scales of the SDQ between 0.41 (peer problems) and 0.67 (hyperactivity–inattention) and for the TDS of 0.80 in British 11–15-year-olds. For Swedish 17–19-year-olds, Svedin and Priebe [13] obtained Cronbach's alpha values for the SDQ scales between 0.50 (conduct problems) and 0.70 (emotional problems and prosocial behavior) and for the TDS of 0.74. Woerner et al. [7] observed reliability coefficients (Cronbach's alpha) in the five scales of the SDQ between 0.20 (peer problems) and 0.65 (hyperactivity–inattention and prosocial behavior) and for the TDS of 0.70 in 11–16-year-olds. Becker et al. [8] reported reliability coefficients (Cronbach's alpha) for the SDQ scales between 0.43 (conduct problems) and 0.64 (hyperactivity–inattention) and for the TDS of 0.72 in German 11–17-year-olds. However, the authors reported higher reliability coefficients, ranging “...between 0.61 (conduct problems) and 0.85 (Total Difficulties score)...” (p. 526), for the same sample when using McDonald's omega. For Spanish adolescents aged 14–19 years, Ortuño-Sierra et al. [14] found reliability coefficients (McDonald's omega) on the five SDQ scales between 0.51 (conduct problems) and 0.71 (emotional problems) and for the TDS of 0.74. Karlsson et al. [11] observed reliability coefficients (Cronbach's alpha) between 0.52 (conduct problems) and 0.76 (hyperactivity–inattention; no information given for the TDS) in Swedish 15–16-year-olds. Bezborodovs et al. [10] found reliability coefficients (ordinal alpha) for 11–15-year-olds between 0.49 (conduct problems) and 0.76 (emotional problems and prosocial behavior) and 0.79 for the TDS for the Latvian language version of the SDQ, as well as between 0.47 (conduct problems) and 0.78 (emotional problems) for the Russian language version, and 0.80 for the TDS.

Regarding the factor structure of the SDQ, different solutions were proposed in representative samples. Originally, Goodman [12] postulated a five-factor structure for SDQ (both for self-assessments and external assessments by parents or teachers) based on an exploratory factor analysis (EFA). Svedin and Priebe [13] first performed a principal component analysis based on self-assessments and obtained a seven-factor solution for the SDQ that explained 52.9% of the total variance (p. 227). The two authors also reported a “...varimax rotated solution restricted to five factors” that explained 44.5% of the variance, which is also mentioned in the abstract of the article [13]. Woerner et al. [7] collected self-assessments from 11–16-year-olds and external assessments from parents and teachers using the SDQ, but only the results of the factor analysis for the parental assessment were reported (a varimax rotation yielded an alternative five-factor solution and explained 42.7% of the variance). According to Becker et al. [8], a five-factor solution showed “...a satisfactory fit to the data...” (p. 523) in German youth. Ortuño-Sierra et al. [14] examined various models in a Spanish sample of adolescents using confirmatory factor analysis (CFA) and, based on the results, described a five-factor solution with correlated errors (p. 4) as the most suitable. Karlsson et al. [11] investigated a total of 16 different models in a Swedish sample of minors with confirmatory factor analyses and concluded from this extensive psychometric examination that “results show a relatively better fit of the 5-factor model compared with the 3-factor model, although neither of these had a satisfactory fit” (p. 1). The authors observed the best fit indices for a five-factor model with cross-loadings and minor factors [11]. Bezborodovs et al. [10] found that the conducted confirmatory factor analyses “... [do] not support the original five-factor or the three-factor structure...” (p. 308). In summary, the findings from representative samples regarding the factor structure of the SDQ in self-assessment are not consistent, and the empirical results on reliability often show low values for some of the scales (e.g., for peer problems and conduct problems), while typically at least acceptable reliability values are reported for the TDS. Further empirical testing, therefore, appears to be necessary with regard to the psychometric properties of the SDQ in self-rating.

In addition to estimating the prevalence of severe mental health problems and evaluating the psychometric properties of the SDQ, in the present representative sample of children and adolescents, it should also be examined whether associations with different types of problematic substance use are evident. In general, the most commonly used psychoactive substances among minors (aged 12–17 years) in Germany are (in order) alcohol, tobacco or other nicotine-containing products, cannabis, ecstasy, and amphetamines [15]. However, a distinction must be made between lifetime prevalence (corresponding to at least one-time use) and regular use or problematic use. For example, for alcohol, in a representative sample of 12–17-year-olds in Germany, the lifetime prevalence was 63.0%, while binge-drinking (a consumption pattern that may be harmful to health) was reported by 15.1% in the last 30 days [15]. Overall, 6.8% of the 12–17-year-olds in Germany described themselves as smokers [15]. Furthermore, in the past 30 days, 6.7% had used disposable e-cigarettes, 3.9% reusable e-cigarettes, and 3.9% hookah [15]. Problematic use of cannabis was reported by 0.4% of the minors in Germany [15]. The use of other illicit drugs among 12–17-year-olds was considerably less common; for example, the 12-month prevalence of amphetamine was 0.4% and of ecstasy 0.3% [15]. According to available empirical findings, the problematic use of alcohol is most common among minors in Germany (e.g., for prevalence estimates in a representative sample, see Wartberg et al. [16]). In another survey, a prevalence of a similar magnitude was found for problematic alcohol use in youth in Germany, along with statistically significant associations between mental health problems (depressive symptoms and conduct problems) [17]. Some longitudinal findings indicate that depressive symptoms [18] and conduct problems [19] may also be important predictors of future problematic alcohol use.

Very few surveys [20],[21] have examined associations between mental health problems (measured using the SDQ) and general substance use (e.g., of alcohol) in representative samples. However, the consumption of alcohol in adolescence is generally a fairly common behavioral pattern, whereas problematic use is much less common but significantly more relevant in terms of future problematic patterns of alcohol use. To our knowledge, relationships between mental health problems and different forms of problematic substance use in representative samples have not yet been investigated in the numerous studies conducted with the SDQ. We investigated the following four research questions (RQ):

RQ1: How high is the prevalence of self-assessed severe mental health problems in the age group 12–17-year-olds in Germany?

RQ2: How reliable is the SDQ (regarding subscales and TDS) in a representative sample of 12–17-year-olds?

RQ3: What results for the factor structure of the SDQ are found in a representative sample of 12–17-year-olds?

RQ4: What are the associations between (severe) mental health problems and problematic use of alcohol, cigarettes, and cannabis in a representative sample of 12–17-year-olds?

## 2. Materials and methods

### 2.1. Procedure

The data collection for the recruitment of a multi-stage random sample of children and adolescents (aged 12–17 years) was carried out by a market research institute by telephone (with a dual-frame design to examine a weighted combined landline and cell phone sample). The response rate (based on completed telephone interviews) was 35.0% (or 4.001 interviews) of the net sample (n = 11.427). Altogether, 35.2% of individuals (n = 4.021) refused to participate, another 25.2% (n = 2.875) could not be reached, and 4.6% (n = 530) abandoned the telephone interview before it was finished. In total, computer-assisted telephone interviews (CATIs) were conducted by 144 trained interviewers with 4001 minors. All participants were informed that all their answers would be stored and evaluated completely anonymously (in the case of 12–13-year-olds, their parents were also informed of this). On average, the entire interview took just about 18 minutes. In order to achieve representativeness for the age group of 12–17-year-olds in Germany, the sample was weighted after completion of the data collection with regard to the following characteristics: gender, year of birth, and region. This was done on the basis of the official population projections of the German Federal Statistical Office. The processes to recruit the participants, to obtain their informed consent, and to collect the data were approved in advance by the local psychological ethics committee at the Center for Psychosocial Medicine of the University Medical Center Hamburg-Eppendorf (LPEK-0196).

### 2.2. Measures

We collected youths' self-ratings of mental health problems and resources in the last six months with the frequently used Strengths and Difficulties Questionnaire (SDQ) [3]. The SDQ comprises 25 items, each with a three-level response format (0 = “Not true”, 1 = “Somewhat true”, 2 = “Certainly true”). Based on the answers to the 25 questions, four problem scales (emotional symptoms, conduct problems, hyperactivity, and peer relationship problems) and one resource scale (prosocial behavior) can be calculated. The four problem scales can be used to compute a Total Difficulties score (TDS, ranging from 0 to 40) [3], considered a good indicator of overall mental health problems [2]. The higher the TDS, the greater the mental health problems. Based on the data of a large, representative epidemiological sample from Germany, Becker et al. [8] recommended a cutoff value ≥17 for the TDS, which was used in the present study.

Problematic alcohol use in the last 12 months was examined with the Alcohol Use Disorders Identification Test (AUDIT) [22]. The AUDIT consists of 10 items. Eight questions have a five-point response format, and the other two items have a three-level response format. A higher sum value over all 10 items (ranging from 0 to 40) points to a higher degree of problematic alcohol consumption. In the present investigation, the reliability coefficient (McDonald's omega) for the AUDIT was 0.74.

Problematic cigarette use was investigated with the Fagerström test for cigarette dependence (FTCD) [23]. The FTCD comprises six questions with an alternating response format. A sum score (range 0–10) can be calculated from the answers. A higher sum value reflects a more intense cigarette dependence. In the sample of the present study, the reliability coefficient (McDonald's omega) for the FTCD was 0.62.

Problematic cannabis use in the past 12 months was measured with the Cannabis Abuse Screening Test (CAST) [24]. The CAST consists of six items with a five-point response format (0 = “never”, 1 = “rarely”, 2 = “from time to time”, 3 = “quite often”, 4 = “very often”). A total score can be computed based on the dichotomized six questions (ranging from 0 to 6). In the present survey, the reliability coefficient (McDonald's omega) for the CAST was 0.80. Furthermore, data on sociodemographic characteristics of the minors (gender, year of birth, migration background, school attendance, and place of residence) were collected.

### 2.3. Statistical analyses

The software IBM SPSS Statistics (Version 27) was used to calculate relative frequencies, reliability coefficients (Cronbach's alpha and McDonald's omega, as recommended, for example, by Hayes and Coutts [25]), correlation analyses, binary logistic regression analyses, and linear regression analyses. Two individuals (or 0.05% of the sample) stated “diverse” when asked about their gender. Since it is not possible to make statistically reliable statements for a group consisting of only two persons, data of these two respondents were not incorporated in further statistical analyses. The dependent variable in both regression models was mental health problems measured with the TDS (for the binary logistic regression analyses as dichotomized, at the suggested cutoff value by Becker et al. [8] for severe mental health problems, and for the linear regression analyses as a dimensional measure). First, we computed bivariate binary logistic regression analyses with the same six explanatory variables (A) gender, (B) age, (C) migration background, (D) problematic alcohol use, (E) problematic cigarette use, and (F) problematic cannabis use. In a second step, we conducted multivariable logistic and linear regression analyses, in which all six explanatory variables were included in both regression models simultaneously.

The statistical software Mplus (Version 8.10) [26] was utilized to conduct confirmatory factor analyses to test a total of four different models: (I) the five-factor model according to Goodman [12], (II) the three-factor model first suggested by Dickey and Blumberg [27], (III) the new five-factor model with correlated errors proposed by Ortuño-Sierra et al. [14], and [IV] the new five-factor model with cross-loadings and minor factors suggested by Karlsson et al. [11].

## 3. Results

### 3.1. Sample description

The representative sample of the present study comprised 4001 children and adolescents in Germany aged between 12 and 17 years. When asked about gender, 48.5% of the total sample (n = 1940) identified as female and 51.5% (n = 2059) as male (0.05%, or two individuals, stated “diverse”). Overall, 21.3% (n = 851) of all respondents reported having a migration background. The majority of children and adolescents (93.0% or n = 3721) still attended school at the time of the survey; approximately four out of five of these young people were in grades 5–10, and the rest were in grades 11–13. Another 6.9% (or n = 278) of individuals were currently no longer attending school (most of them were in vocational training), while data on this feature were lacking for 0.05% (or n = 2) of the sample.

### 3.2. Prevalence estimation for severe mental health problems

Answer to research question 1 (RQ1): According to the cutoff point (≥17 points) suggested by Becker et al. [8] for the TDS for self-assessment of the German version of the SDQ, 9.6% (95% confidence interval: 8.7%–10.5%) of the surveyed sample reported severe mental health problems (see Table 1 for this result in the present study and available findings from other representative samples).

### 3.3. Reliability analyses

Answer to research question 2 (RQ2): Based on Cronbach's alpha, we observed reliability values for the five scales of the SDQ between 0.44 (peer problems) and 0.70 (emotional problems) and, based on McDonald's omega, between 0.45 (peer problems) and 0.70 (emotional problems). The values for the TDS were 0.75 (Cronbach's alpha) and 0.74 (McDonald's omega). All reliability values can be found in Table 2.

**Table 1.** Prevalence estimates of severe mental health problems in representative samples based on self-ratings for the Total Difficulties score (TDS) of the SDQ.

Studies	Van Roy et al. (2008) Norway	Woerner et al. (2011) Thailand	Becker et al. (2018) Germany	Gray et al. (2021) Australia	Bezborodovs et al. (2024) Latvia	Wartberg et al. (2026) Germany
Severe mental health problems (cutoff value)	8.7% (≥19)	8.9% (≥19)	8.8% (≥17)	10.2% (≥17)	9.4%^1^ / 8.7%^2^ (≥22)	9.6% (≥17)

Note: ^1^ Latvian language version. ^2^ Russian language version.

**Table 2.** Reliability coefficients for the subscales and the Total Difficulties score of the SDQ.

Scale	Cronbach's alpha	McDonald's omega
Emotional problems	0.70	0.70
Conduct problems	0.48	0.49
Hyperactivity–inattention	0.66	0.67
Peer problems	0.44	0.45
Prosocial behavior	0.49	0.50
TDS	0.75	0.74

**Table 3.** Goodness-of-fit indices of the confirmatory factor analyses of the SDQ in a representative sample.

Models	CFI	TLI	RMSEA	SRMR
Five-factor model^1^	0.84	0.82	0.04	0.04
Three-factor model^2^	0.79	0.76	0.05	0.05
Five-factor model with correlated errors^3^	0.89	0.88	0.04	0.03
Five-factor model with cross-loadings and minor factors^4^	0.89	0.87	0.04	0.03

Note: ^1^ Proposed by Goodman [12]. ^2^ Proposed by Dickey and Blumberg [25]. ^3^ Proposed by Ortuño-Sierra et al. [14]. ^4^ Proposed by Karlsson et al. [11].

### 3.4. Confirmatory factor analyses

Answer to research question 3 (RQ3): The results of the four confirmatory factor analyses (CFAs) are presented in Table 3. The relatively best fit when comparing the four models was found for the five-factor model with correlated errors suggested by Ortuño-Sierra et al. [14]; however, no model showed a completely satisfactory fit to the data.

### 3.5. Correlation and regression analyses

Answer to research question 4 (RQ4): In bivariate and multivariable analyses, we consistently found statistically significant associations between mental health problems (measured with the TDS) and problematic cigarette use and problematic cannabis use. These results were shown in Table 4, Table 5 and Table 6. The results for problematic cigarette use and problematic cannabis use were also evident when controlling for gender, age (measured via year of birth), and migration background in both multivariable analyses. Problematic alcohol use was found only in the (bivariate) correlation analyses and the multivariable linear regression analyses related to mental health problems, but not in the binary logistic regression analyses.

**Table 4.** Associations between mental health problems, sociodemographic aspects, and problematic use of alcohol, cannabis, and cigarettes in the binary logistic regression analyses.

Variable	Mental health problems, bivariate associations Odds ratios (95% confidence interval)	Mental health problems, multivariable associations Adjusted odds ratios (95% confidence interval)
Gender^a^	2.16*** (1.73;2.70)	2.25*** (1.80;2.82)
Year of birth^b^	1.01 (0.95;1.07)	1.03 (0.96;1.11)
Migration background^c^	1.47** (1.16;1.87)	1.54*** (1.21;1.97)
Problematic alcohol use	1.02 (0.99;1.05)	1.00 (0.96;1.04)
Problematic cigarette use	1.42*** (1.22;1.64)	1.36*** (1.14;1.60)
Problematic cannabis use	1.44*** (1.23;1.69)	1.26* (1.03;1.54)

Note: ^a^ coding: 0 = male, 1 = female. ^b^ the higher the year of birth, the lower the age. ^c^ coding: 0 = no migration background, 1 = migration background. ***p < 0.001, **p < 0.010, *p < 0.050.

**Table 5.** Bivariate associations in the correlation matrix for the variables.

Variable	1	2	3	4	5	6	7
(1) Gender^a^	–						
(2) Year of birth^b^	0.00	–					
(3) Migration background^c^	−0.04*	0.01	–				
(4) Mental health problems^d^	0.16***	−0.02	0.06***	–			
(5) Problematic alcohol use	−0.04*	−0.52***	−0.08***	0.09***	–		
(6) Problematic cigarette use	−0.03	−0.14***	0.01	0.10***	0.26***	–	
(7) Problematic cannabis use	−0.01	−0.14***	−0.01	0.12***	0.34***	0.30***	–

Note: ^a^ coding: 0 = male, 1 = female. ^b^ the higher the year of birth, the lower the age. ^c^ coding: 0 = no migration background, 1 = migration background. ^d^ TDS of the Strengths and Difficulties Questionnaire. ***p < 0.001, **p < 0.010, *p < 0.050.

**Table 6.** Associations between mental health problems, sociodemographic aspects, and problematic use of alcohol, cannabis, and cigarettes in the linear regression analyses.

Variable	Mental health problems, multivariable associations Adjusted Beta coefficients (95% confidence interval)
Gender^a^	0.17*** (0.13;0.19)
Year of birth^b^	0.04* (0.00;0.07)
Migration background^c^	0.07*** (0.04;0.10)
Problematic alcohol use	0.07*** (0.03;0.11)
Problematic cigarette use	0.07*** (0.04;0.10)
Problematic cannabis use	0.08*** (0.04;0.11)

Note: ^a^ coding: 0 = male, 1 = female. ^b^ the higher the year of birth, the lower the age. ^c^ coding: 0 = no migration background, 1 = migration background. ***p < 0.001, **p < 0.010, *p < 0.050.

## 4. Discussion

The aim of this study was to determine the prevalence of severe mental health problems in a large representative sample of children and adolescents and to determine the associations with problematic substance use, as well as to examine the psychometric properties of an internationally widely used instrument (Strengths and Difficulties Questionnaire, SDQ [3]) for self-assessed mental health problems and prosocial behavior. The estimated prevalence of 9.6% for severe mental health problems among 12–17-year-olds in Germany (based on the TDS) was within the range of published values from other representative samples. The lowest prevalence known to us, according to the TDS (8.7%), in a representative sample, was found in the survey by Van Roy et al. [6] for Norwegian 10–19-year-olds, while the highest prevalence estimate (10.2%) was observed by Gray et al. [9] for 11–17-year-olds from Australia. In direct comparison with the value of 8.8% for severe mental health problems obtained by Becker et al. [8], which was also determined for a representative sample in Germany, the present study showed the numerically higher value 9.6% (see Table 1). Whether this actually represents an increase in the prevalence of self-rated severe mental health problems among German children and adolescents is unclear, as the 95% confidence interval determined in our survey (8.7%–10.5%) includes the value reported by Becker et al. [8]. The values reported by Van Roy et al. [6] and Gray et al. [9] also fall within this range. However, all comparisons must take into account the different age ranges of the samples investigated and, above all, the different cutoff values used for the TDS of SDQ (in the present study, the cutoff value of ≥17 recommended for Germany by Becker et al. [8] was utilized). In the survey by Gray et al. [9] from Australia, the same cutoff value (≥17) was used for TDS, and the empirical findings suggest a prevalence of severe mental health problems in German and Australian children and adolescents of a similar magnitude. In the future, it would be desirable to regularly record (e.g., every few years in a country) self-rated severe mental health problems, so that changes can be detected more quickly and countermeasures taken if necessary (e.g., in the event of an increase in prevalence, with an expansion of preventive approaches).

Regarding the psychometric properties of the widely used SDQ, this study reveals some findings that are similar to those of other representative samples for self-assessment in the instrument, although there are also some differences. In scientific literature, a value of 0.70 is often cited as still acceptable for reliability (e.g., [28]), whereas Aiken and Groth-Marnat [29] recommended a value of 0.60 in group studies as a lower limit for a reliability value. Based on these recommendations for lower limits for sufficient reliability, only *emotional problems* and *hyperactivity* were above these values at the scale level in the present study, while the SDQ scales *conduct problems*, *peer problems*, and *prosocial behavior* were clearly below (see Table 2). Such problems with regard to reliability had already been observed on several occasions in other representative samples for self-ratings in SDQ, for example, for *conduct problems* [7],[10],[11],[13],[14], *peer problems* [7],[11]–[14], and *prosocial behavior* [14]. In the investigation by Bezborodovs et al. [10], reliability values <0.6 on the *hyperactivity* scale were also observed (at least for the Latvian-language version of the SDQ). Unlike what Becker et al. [8] reported for their representative sample, in the present study, the use of McDonald's omega instead of Cronbach's alpha to determine reliability changed the values only very minimally (see also Table 2). While these empirical findings mean that the use of individual SDQ scales concerning reliability can certainly be critically questioned, acceptable-to-good parameters were reported consistently across the studies for TDS (as the sum of four scales). The use of TDS (which was used as the central measure of SDQ in this survey) can therefore also be recommended in self-assessment with regard to reliability.

Concerning the factor structure of the SDQ, clear differences were found in representative samples between the results of exploratory and confirmatory factor analyses. The results of the confirmatory factor analyses in the present study showed that neither the proposed three-factor model nor the five-factor model had a completely satisfactory fit to the data (rather an approximation to fit indices that some authors describe as “acceptable”, e.g., [30]), even taking into account various modifications of this model, such as correlated errors (model with the best parameters in the representative sample from Ortuño-Sierra et al. [14]) or cross-loadings and minor factors (model with the best parameters in the representative sample from Karlsson et al. [11]). There may be differences between SDQ self-assessments and external assessments in terms of factor structure. For example, in a systematic review by Kersten et al. [31], better fit indices were reported for factor analyses based on teacher assessments in the SDQ. In view of the inconsistent results in the factorial structure, it would be worth considering whether and what modifications to the self-report version of the SDQ could improve this; meanwhile, some researchers are suggesting a revision of the instrument [32].

Data from representative samples on associations between several different forms of problematic substance use and mental health problems (recorded with the SDQ) were not yet available (although numerous studies had been conducted with the instrument). The findings of the present survey complement the literature in that both the problematic use of cannabis (measured with the CAST [24]) and of cigarettes (assessed with the FTCD [23]) are associated with increased mental health problems (evident in both the categorical and dimensional evaluation approaches and also when adjusting for sociodemographic aspects such as gender and age). For problematic alcohol use (recorded with the AUDIT [22]), however, findings were not consistent. While statistically significant relations were found in the dimensional analysis (also in the multivariable model), these findings did not emerge from the categorical evaluation approach. Fidalgo et al. [21] found consistent relationships between higher TDS and general use and regular consumption (in the last month) of alcohol, tobacco, and marijuana. However, general consumption or regular use of a substance in the last month is not equivalent to problematic use or an addictive disorder (which, according to ICD-10 [33], for example, should typically be assessed over a period of 12 months). There is clearly a need for further research, particularly in representative samples of adolescents, for example, in longitudinal studies, to assess if and to what extent the problematic use of cannabis or cigarettes also predicts increased mental health problems. In addition to substance-related disorders, in the ICD-11, “Disorders due to addictive behaviors” (non-substance-related disorders, for example [34]) were also included, and longitudinal examinations on relationships with mental health problems in representative samples would also be interesting.

The present study has several limitations. For example, only self-assessments of children and adolescents were collected in the SDQ; assessments by parents or even teachers would have been interesting additions (combined designs for different age groups were utilized, for example, by Bunting et al. [35]). On the one hand, external ratings for mental health problems are well established in these age groups (with the SDQ or, for example, with the more comprehensive Child Behavior Checklist [36]). On the other hand, external assessments of adolescent problematic substance use are uncommon, and it is therefore unclear to what extent such information (e.g., by parents) would be valid. Furthermore, the SDQ assesses the last six months; whether the mental health problems of surveyed children and adolescents are temporary or longer lasting cannot be conclusively assessed on the basis of this data. In the present study, mental health problems were self-assessed, which is not equivalent to diagnoses from the ICD-10 [33] or according to ICD-10, which are made by experts and considered to be of higher methodological quality. Besides, with regard to regular use of cannabis, empirical findings are available (including among adolescents from Germany [37]), indicating cognitive problems that could potentially influence response behavior (e.g., in surveys). However, the findings on such difficulties are not consistent (see, for example, the review by Blest-Hopley et al. [38]). Data for this representative sample were collected in Germany. Concerning the consumption of psychotropic substances, legal regulations are also relevant, as well as social attitudes toward substance use; for example, in Germany, the purchase and consumption of certain alcoholic beverages, such as beer, wine, or sparkling wine, is legal from the age of 16. These aspects should be taken into account when interpreting the findings and considering their transferability to other countries or cultural circles. This study is a cross-sectional survey, which means that the associations cannot be interpreted causally. Whether problematic substance use promotes more mental health problems or vice versa is unclear and cannot be determined with this type of study design. Longitudinal research designs would be necessary to determine causal relationships.

## 5. Conclusions

Despite the limitations mentioned, the present study yielded interesting results. In addition to a more recent prevalence estimate for severe mental health problems for Germany than the one presented by Becker et al. [8], the significance of different types of problematic substance use for mental health problems at an early age has already been demonstrated in a large representative sample. According to the findings of this survey, a substantial proportion of German children and adolescents are affected by severe mental health problems. Both in primary and secondary prevention measures, as well as in interventions for minors, problematic substance use (especially of cannabis or cigarettes) should be considered as a possible source of mental health problems (e.g., in the form of a standardized assessment in clinical settings). The co-occurrence of mental health problems and problematic substance use also has practical implications that should be taken into account in treatment (see, for example, Fernández-Artamendi et al. [39]).

## Use of AI tools declaration

The authors declare they have not used Artificial Intelligence (AI) tools in the creation of this article.

## References

[1] Kieling C, Baker-Henningham H, Belfer M (2011). Child and adolescent mental health worldwide: evidence for action. Lancet.

[2] Goodman A, Goodman R (2009). Strengths and difficulties questionnaire as a dimensional measure of child mental health. J Am Acad Child Adolesc Psychiatry.

[3] Malmberg M, Rydell AM, Smedje H (2003). Validity of the Swedish version of the Strengths and Difficulties Questionnaire (SDQ-Swe). Nord J Psychiatry.

[4] Goodman R (1997). The strengths and difficulties questionnaire: a research note. J Child Psychol Psychiatry.

[5] Goodman R, Meltzer H, Bailey V (1998). The strengths and difficulties questionnaire: A pilot study on the validity of the self-report version. Eur Child Adolesc Psychiatry.

[6] Van Roy B, Grøholt B, Heyerdahl S (2006). Self-reported strengths and difficulties in a large Norwegian population 10–19 years: age and gender specific results of the extended SDQ-questionnaire. Eur Child Adolesc Psychiatry.

[7] Woerner W, Nuanmanee S, Becker A (2011). Normative data and psychometric properties of the Thai version of the Strengths and Difficulties Questionnaire (SDQ). J Ment Health Thai.

[8] Becker A, Wang B, Kunze B (2018). Normative data of the self-report version of the German strengths and difficulties questionnaire in an epidemiological setting. Z Kinder Jugendpsychiatr Psychother.

[9] Gray EJ, Scott JG, Lawrence DM (2021). Concordance between adolescents and parents on the Strengths and Difficulties Questionnaire: Analysis of an Australian nationally representative sample. Aust N Z J Psychiatry.

[10] Bezborodovs Ņ, Krēgers R, Vētra L (2024). Psychometric properties and normative data of the Latvian and Russian language versions of the strengths and difficulties questionnaire (SDQ) in the Latvian general adolescent population. Nord J Psychiatry.

[11] Karlsson P, Larm P, Svensson J (2022). The factor structure of the strength and difficulties questionnaire in a national sample of Swedish adolescents: Comparing 3 and 5-factor models. PLoS One.

[12] Goodman R (2001). Psychometric properties of the strengths and difficulties questionnaire. J Am Acad Child Adolesc Psychiatry.

[13] Svedin CG, Priebe G (2008). The strengths and difficulties questionnaire as a screening instrument in a community sample of high school seniors in Sweden. Nord J Psychiatry.

[14] Ortuño-Sierra J, Sebastián-Enesco C, Pérez-Albéniz A (2022). Spanish normative data of the Strengths and Difficulties Questionnaire in a community-based sample of adolescents. Int J Clin Health Psychol.

[15] Orth B, Spille-Merkel C, Nitzsche A (2025). Die Drogenaffinität Jugendlicher in der Bundesrepublik Deutschland 2023, Substanzkonsum und Internetnutzung im Jahr 2023 und Trends.

[16] Wartberg L, Kriston L, Thomasius R (2019). Prevalence of problem drinking and associated factors in a representative German sample of adolescents and young adults. J Public Health.

[17] Wartberg L, Brunner R, Kriston L (2016). Psychopathological factors associated with problematic alcohol and problematic Internet use in a sample of adolescents in Germany. Psychiatry Res.

[18] Skogen JC, Knudsen AK, Hysing M (2016). Trajectories of alcohol use and association with symptoms of depression from early to late adolescence: The Norwegian longitudinal health behaviour study. Drug Alcohol Rev.

[19] Wartberg L, Fischer-Waldschmidt G, Kriston L (2021). Longitudinal predictors of problematic alcohol use in adolescence: A 2-year follow-up study. Addict Behav.

[20] Goodman A (2010). Substance use and common child mental health problems: examining longitudinal associations in a British sample. Addiction.

[21] Fidalgo TM, Sanchez ZM, Caetano SC (2016). The association of psychiatric symptomatology with patterns of alcohol, tobacco, and marijuana use among Brazilian high school students. Am J Addict.

[22] Babor TF, Higgins-Biddle JC, Saunders JB (2001). World Health Organization: AUDIT: The Alcohol Use Disorders Identification Test: guidelines for use in primary care.

[23] Fagerström K (2011). Determinants of tobacco use and renaming the FTND to the Fagerström test for cigarette dependence. Nicotine Tob Res.

[24] Legleye S, Karila L, Beck F (2007). Validation of the CAST, a general population cannabis abuse screening test. J Subst Use.

[25] Hayes AF, Coutts JJ (2020). Use omega rather than Cronbach's alpha for estimating reliability. But.... Commun Methods Meas.

[26] Muthen LK, Muthen BO (2017). Mplus user's guide.

[27] Dickey WC, Blumberg SJ (2004). Revisiting the factor structure of the strengths and difficulties questionnaire: United States, 2001. J Am Acad Child Adolesc Psychiatry.

[28] Danner D (2015). Reliability – the accuracy of a measurement. Reliabilität – die Genauigkeit einer Messung.

[29] Aiken LR, Groth-Marnat G (2006). Psychological Testing and Assessment.

[30] Marsh HW, Hau KT, Wen Z (2004). In search of golden rules: Comment on hypothesis-testing approaches to setting cutoff values for fit indexes and dangers in overgeneralizing Hu and Bentler's (1999) findings. Struct Equ Modeling.

[31] Kersten P, Czuba K, McPherson K (2016). A systematic review of evidence for the psychometric properties of the Strengths and Difficulties Questionnaire. Int J Behav Dev.

[32] Kankaanpää R, Töttö P, Punamäki RL (2023). Is it time to revise the SDQ? The psychometric evaluation of the Strengths and Difficulties Questionnaire. Psychol Assessment.

[33] Dilling H, Freyberger H (2019). Pocket guide to the ICD-10 classification of mental disorders. Taschenführer zur ICD-10-Klassifikation psychischer Störungen.

[34] Wartberg L, Zitzmann S, Diestelkamp S (2025). Problematic use of video games, social media, and alcohol: Exploring reciprocal relations with the big five personality traits in a longitudinal design. Eur J Investig Health Psychol Educ.

[35] Bunting L, McCartan C, Davidson G (2022). Rationale and methods of the ‘Northern Ireland Youth Wellbeing Survey’ and initial findings from the Strengths and Difficulties Questionnaire. Clin Child Psychol Psychiatry.

[36] Achenbach TM, Becker A, Döpfner M (2008). Multicultural assessment of child and adolescent psychopathology with ASEBA and SDQ instruments: research findings, applications, and future directions. J Child Psychol Psychiatry.

[37] Wartberg L, Thomsen M, Thomasius R (2014). Gedächtnis- und Aufmerksamkeitsfunktionen bei Jugendlichen mit intensivem regelmäßigem Cannabiskonsum. Z Neuropsychol.

[38] Blest-Hopley G, Colizzi M, Giampietro V (2020). Is the adolescent brain at greater vulnerability to the effects of cannabis? A narrative review of the evidence. Front Psychiatry.

[39] Fernández-Artamendi S, Flórez Menéndez G, Cortés Tomás MT (2024). Dual pathology? Reviewing the conceptualization of comorbidity in addictions. Adicciones.

